# Structural Properties and Anti-Inflammatory Activity of GLP-P, a Kefir-Derived Neutral Glycopeptide

**DOI:** 10.3390/foods14203509

**Published:** 2025-10-15

**Authors:** Yuejiao Yang, Zhiying Zhang, Ying Bai

**Affiliations:** Laboratory National Characteristic Food Research and Development, Inner Mongolia Agricultural University, Hohhot 010018, China; yangyuejiao150@163.com (Y.Y.);

**Keywords:** kefir, glycopeptide, structure, anti-inflammatory activity, NF-κB

## Abstract

Bioactive glycopeptides, commonly present in natural foods, exhibit notable immunomodulatory and neuroprotective effects. However, naturally occurring low-molecular-weight glycopeptides have rarely been reported, and their structural and functional properties remain insufficiently explored. In this study, a low-molecular-weight glycopeptide complex (GLP-P) was isolated from kefir. The structure of GLP-P was characterized via molecular weight (Mw) determination, monosaccharide and amino acid composition analysis, Fourier transform infrared spectroscopy, methylation analysis, and nuclear magnetic resonance spectroscopy. GLP-P had a molecular weight of 1192 Da and mainly consisted of four monosaccharides (glucose 64.7%, galactose 34.4%, and others 2.0%) and eight amino acids (asparagine 30.89 ± 0.01 μg/mg, threonine 8.71 ± 0.04 μg/mg, serine 9.5 ±0.08 μg/mg and others.). The primary chain structure of GLP-P contained β-D-Galp-(1→4)-β-D-Glcp-(1→4)-α/β-D-Glcp linkages, including both α- and β-D-Glcp isomers. Notably, the HMBC spectrum of GLP-P exhibits putative O-glycosylation characteristics. Functionally, GLP-P significantly reduced IL-6 and TNF-α expression while enhancing IL-10 in LPS-stimulated RAW264.7 macrophages. These effects were associated with inhibition of the TLR4/NF-κB pathway. These findings suggest that low-molecular-weight GLP-P has shown potential efficacy in the treatment of inflammation in vitro. These results provide a theoretical basis for kefir glycopeptide development of functional foods and as an adjuvant therapeutic agent for inflammation-related disorders in the future.

## 1. Introduction

Kefir is a fermented milk product with a unique flavor and rich nutritional profile made from fermentation with kefir grains, which are complex, milky white, gelatinous, cauliflower-like, and popcorn-shaped. These kefir grains are formed via the adhesion of various probiotics such as lactic acid bacteria, yeasts, and acetic acid bacteria within an extracellular matrix [1,2]. Milk fermented with kefir grains not only has the characteristic sour and aromatic flavor of fermented milk but also has a slight alcoholic aroma and natural effervescence. This healthy sour and viscous beverage is popular in many regions [3], and several studies have shown that its regular consumption can alleviate inflammation in the body [4] and regulate gut microbiota [5]. Furthermore, the regular consumption of kefir has also been linked to improved lipid profiles and a lower risk of obesity [6].From a therapeutic perspective, kefir has shown some effectiveness in the management of osteoporosis [7] and potential in delaying neurodegenerative processes. [8]. It has also demonstrated a wide range of nutritional benefits and bioactivities, including antioxidant [9], antitumor, and antihypertensive effects [10]. Typically, these biological activities are the result of the bioactive peptides, polysaccharides, amino acids, organic acids, and other bioactive substances produced by microorganisms during kefir fermentation and storage [11,12].

Glycopeptide is defined as a complex formed through covalent or non-covalent interactions between glycans and peptides chain. Naturally occurring glycopeptides are widely distributed in terrestrial animals, marine organisms, plants, and microorganisms, and can be effectively concentrated and purified by hydrolysis and chromatographic techniques [13]. Accumulating evidence indicates that bioactive glycopeptides possess considerable potential in anti-inflammatory [14], anti-tumor [15], and anti-viral activities [16], as well as in the alleviating of cardiovascular [17] and delaying neurodegenerative diseases [18]. In addition, they have shown notable functional advantages in food processing applications [19]. These findings have garnered considerable attention in the fields of food science and health applications. Kefir fermented milk, which contains abundant protein and carbohydrate substrates [20] together with a complex microbial enzymatic system [21,22], represents a reliable source for the extraction of glycopeptide complexes.

Current research on kefir metabolites has primarily focused on polysaccharides and peptides [23,24], whereas reports on kefir-derived glycopeptides remain limited. To the best of our knowledge, this study is the first to isolate and characterize glycopeptides from kefir and to explore their potential anti-inflammatory activity. Specifically, GLP-P was extracted from fermented whey derived from kefir grain–fermented skimmed milk powder, and its structure was elucidated using a series of analytical techniques. In addition, the possible anti-inflammatory effects of GLP-P were investigated in an LPS-induced RAW264.7 macrophage model. Taken together, this comprehensive analysis provides preliminary evidence and a theoretical basis for the further development of kefir-derived functional products with potential health benefits.

## 2. Materials and Methods

### 2.1. Reagents and Materials

Herders from Xilingol League in China’s Inner Mongolia Autonomous Region supplied the kefir grain. RAW264.7 cells were obtained from Wuhan HUAYAN Biotechnology Co., Ltd. (Wuhan, Hubei, China). Skimmed milk powder was obtained from Alar Foods amba (Vimmerby, Sweden). All other reagents used in the study were of analytical grade.

### 2.2. Extraction, Separation, and Purification of GLP-P

In an aseptic environment, kefir grains were inoculated at 5% (*w*/*v*) into a 10% (*w*/*v*) skim milk solution (500 mL) and fermented at constant temperature 28 °C without stirring for 36 h (pH = 3.8 ± 0.1), fermentation was performed in three independent batches in parallel. The supernatant was collected by centrifugation at 10,950× *g* for 15 min, followed by the addition of two volumes of cold ethanol and precipitation at 4 °C for 24 h. The resulting precipitate was recovered by centrifugation at 10,000 rpm for 10 min, resuspended in 10 volumes of ultrapure water (*w*/*v*), and centrifugation at 10,950× *g* for 15 min again. The clear supernatant was freeze-dried to obtain crude kefir glycopeptides (GLP), with a yield of 1.08 ± 0.08 g/L. The crude GLP was dissolved in water and subjected to preliminary purification on a DEAE-Sepharose Fast Flow column (Boruitang, Yangzhou, China) (1 cm × 50 cm) at a flow rate of 1 mL/min with stepwise elution using water, 0.2 M NaCl, 0.5 M NaCl, and 1 M NaCl solutions [25]. Eluates were collected every 5 min per tube, and the water-eluted fraction was concentrated and freeze-dried to obtain the neutral polysaccharide fraction. Further purification was performed using a Sephadex G-100 gel filtration column (Boruitang, Yangzhou, China) with distilled water as the mobile phase (0.5 mL/min), yielding the purified glycopeptide complex fraction GLP-P. The extraction rate (R) was calculated using the following Formula (1):

(1) $$R\;(\%)\;=\frac{{\mathrm{Weight}}_{\mathrm{GLP-P}}}{{\mathrm{Weight}}_{\mathrm{GLP}}}\times 100,$$

### 2.3. Structural Identification

All structural analyses described below were performed by Yangzhou Boruitang Company (Yangzhou, China) for testing purposes.

#### 2.3.1. Molecular Weight Measurement

The molecular weight of GLP-P was determined using a Thermo U3000 system (Thermo, Waltham, MA, USA) equipped with a BRT 105–103–101 gel filtration column (Borutang, Yangzhou, China) (8 mm × 300 mm). Chromatographic parameters were as follows: column temperature: 40 °C; injection volume: 100 μL; mobile phase: 0.5 M NaCl; flow rate: 0.7 mL/min [26]. The manufacturer information for the molecular weight standards is provided in Table S1, and the details of the molecular weight calibration curve are presented in Table S1 and Figure S2.

#### 2.3.2. Monosaccharide Composition Analysis

The GLP-P was pretreated using the previously described method of Shan [27]. Ion chromatography was used to analyze the resultant supernatant.

The Dionex Carbopac ™ PA10 column (4 mm × 250 mm) (Thermo, Waltham, MA, USA) was used for gradient elution separation of monosaccharides. The mobile phases were: (A) ultrapure water, (B) 500 mM NaOH + 50 mM sodium acetate, (C) 20 mM NaOH. Elution parameters were as follows: flow rate: 1.0 mL per min; column temperature: 30 °C; UV detection wavelength: 250 nm; gradient program (0–30 min, 0–50% A and 100–50% C; 30–30.1 min, 50–70% A, 0–30% B, 50–0% C; 30.1–46 min, 70% A and 30% B). Monosaccharide identification and quantification were performed using external standards, as listed in Table S3. The manufacturer information for the Monosaccharide Composition standards is provided in Table S4.

#### 2.3.3. Amino Acid Composition Analysis

GLP-P (5 mg) was hydrolyzed in 2 mL of 6 M hydrochloric acid at 100 °C for 8 h. The hydrolysate was dried under a stream of nitrogen and then processed according to the procedure described in Section 2.3.2. Amino acid composition was analyzed using a Dionex AminoPac™ PA10 column (2 mm × 50 mm) (Thermo, Waltham, MA, USA) with gradient elution. The mobile phase consisted of (A) ultrapure water, (B) 200 mM NaOH, and (C) 25 mM NaOH + 1 M sodium acetate. Detection was performed by pulsed amperometric detection (PAD) using an electrochemical detector. Elution parameters were as follows: column temperature, 30 °C; injection volume, 25 µL; and flow rate, 0.25 mL/min. Amino acids were identified and quantified using a standard mixture (Table S5). The manufacturer information for the amino acid sample standards is provided in Table S6.

#### 2.3.4. Fourier Transform Infrared Spectroscopy

The Fourier transform infrared (FTIR) spectrum of GLP-P was recorded using a Thermo Fisher Scientific iS10 spectrometer (Thermo, Waltham, MA, USA) over the range of 400–4000 cm^−1^. Baseline correction, deconvolution, and second-derivative peak fitting of the amide I band (1600–1700 cm^−1^) were performed using PeakFit 4.12 software to calculate the relative proportions of secondary structural elements in the sample.

#### 2.3.5. Nuclear Magnetic Resonance

Nuclear magnetic resonance (NMR) analysis of GLP-P was performed according to the method described by Feng [28]. Briefly, 50 mg of GLP-P was dissolved in 0.5 mL of D_2_O (99.9%) and subjected to three cycles of freeze-drying and redissolution to ensure complete exchange of labile protons. The ^1^H NMR, ^13^C NMR, DEPT-135, and 2D NMR spectra were acquired at 25 °C on a 600 MHz NMR spectrometer.

#### 2.3.6. Atomic Force Microscope (AFM)

GLP-P was dissolved in ethanol (20 μg/mL). A 10 μL aliquot of the solution was dropped onto a mica sheet and air-dried (25 °C, 12 h). AFM imaging was performed in tapping mode using a Bruker ICON atomic force microscope (Bruker, Billerica, MA, USA). The scanning area was set to 5 × 5 μm.

### 2.4. Cell Experiments

The cell assay measurements were performed by Wuhan Huayan Biotechnology Company (Wuhan, Hubei, China).

#### 2.4.1. Cell Culture

RAW264.7 cells were cultured according to the method described by Phan [29]. The cells were maintained in high-glucose DMEM supplemented with 10% FBS (Gibco, San Francisco, CA, USA) and 1% penicillin–streptomycin (P/S), at 37 °C in a humidified atmosphere containing 5% CO_2_.

#### 2.4.2. Cell Viability Assay

The method for cell viability determination was based on reference [30]. RAW264.7 cells in the logarithmic growth phase were seeded into 96-well plates at a density of 1.8 × 10^4^ cells per well. The cells were treated with GLP-P (50, 200, and 800 μg/mL) or an equal volume of DMEM (control group) for 24 h. Cell viability was assessed using a CCK-8 cell proliferation and cytotoxicity assay kit (Nanjing Jiancheng, Nanjing, China). The calculation formula was as follows (2):

(2) $$\mathrm{Cell}\;\mathrm{viability}\;(\%)\;=\frac{A_{\mathrm{experimental}}-A_{\mathrm{blank}\;}}{A_{\mathrm{control}}-A_{\mathrm{blank}\;}}\times 100,$$

#### 2.4.3. Scanning Electron Microscopy (SEM)

Cell morphology was examined using a Hitachi SU8100 scanning electron microscope (Hitachi, Tokyo, Japan). Images were acquired at an accelerating voltage of 3.0 kV with a magnification of 500×.

#### 2.4.4. NO Measurement

Cells were pretreated with GLP-P (50, 200, or 800 μg/mL) or aspirin (1 μg/mL), followed by stimulation with lipopolysaccharide (LPS, *Escherichia coli* O111:B4; 1 μg/mL). The cells were then co-cultured for 24 h as described in Section 2.4.1. Nitric oxide (NO) release levels were subsequently measured in each group using a commercial NO detection kit (Nanjing Jiancheng, Nanjing, China).

#### 2.4.5. Enzyme-Linked Immunosorbent Assay (ELISA)

The concentrations of inflammatory cytokines were determined using commercial ELISA kits (Meimian, Jiangsu, China) according to the manufacturer’s instructions. Cytokine levels were calculated based on the corresponding standard curves, and the kit model numbers are provided in Table S7.

#### 2.4.6. Real-Time Quantitative PCR (RT-qPCR)

The specific experimental procedure is slightly modified based on the research report by Zeng [31]. The specific primers are detailed in Table S8. Real-time qRT-PCR analysis was conducted on an ABI QuantStudio 6 Real-Time PCR System (Applied Biosystems, Foster City, CA, USA) under the following conditions: 95 °C for 10 min, 40 cycles of 95 °C for 15 s, 60 °C for 60 s and 95 °C for 15 s, and a melting curve from 60 °C for 60 s to 95 °C for 15 s to ensure amplification of a single product. The GADPH was amplified as a corresponding control in all samples to represent the housekeeper gene.

#### 2.4.7. Western Blotting Analysis

Cells were lysed in RIPA buffer (Meilunbio, Dalian, China) containing a phosphatase inhibitor (Meilunbio, Dalian, China), and protein concentrations were determined using a BCA protein assay kit. Equal amounts of protein (20 μg) were separated by SDS-PAGE on polyacrylamide gels and subsequently transferred onto polyvinylidene difluoride (PVDF) membranes [32].

The membranes were blocked in TBST containing 5% skimmed milk, while phosphorylated proteins were blocked with 1% BSA, at room temperature for 2 h on a shaker to ensure adequate contact with the buffer. The membranes were then incubated with primary antibodies overnight at 4 °C (anti-P65, 1:2000; anti-GAPDH, anti-p-P65, anti-IκB, anti-p-IκB, anti-TLR4, anti-MyD88, anti-IKKβ, and anti-p-IKKβ, all at 1:1000), followed by incubation with HRP-conjugated secondary antibodies (1:10,000) for 2 h at room temperature. Protein bands were visualized using an enhanced chemiluminescence (ECL) detection kit, and band intensities were analyzed by Image-Pro Plus 6.0 software. GAPDH was used as the loading control. The manufacturer information for the antibodies is provided in Table S9.

#### 2.4.8. Immunofluorescence Staining

The fixation procedure was performed according to the method of Sun [33] with slight modifications. Cells grown on glass coverslips were fixed with 4% paraformaldehyde for 15 min, followed by three washes with phosphate-buffered saline (PBS). The cells were then permeabilized with 0.5% Triton X-100 (prepared in PBS) for 20 min and blocked with normal goat serum (Biosharp, Hefei, China) for 30 min. Subsequently, the cells were incubated with a 1:100 dilution of the anti-p65 primary antibody at 4 °C for 12 h, followed by incubation with a 1:400 dilution of the sheep anti-rabbit IgG secondary antibody at 37 °C for 1 h (Thermo Fisher Scientific, USA). The nuclei were counterstained with DAPI for 5 min, and all staining steps were performed in the dark [34]. Finally, the cells were mounted with an anti-fade mounting medium and imaged using a Nikon C2 fluorescence microscope (Nikon, Tokyo, Japan). The manufacturer information for the fluorescent immunoassay reagents is provided in Table S10.

### 2.5. Statistical Analysis

All data are presented as the mean ± SD from three independent experiments. The data were analyzed using SPSS Statistics 26 software. Specifically, one-way analysis of variance (ANOVA) followed by Tukey’s post hoc test was employed to assess significant differences, with *p* < 0.05 considered statistically significant.

## 3. Results

### 3.1. Gel Column Separation and Purification Results

**Figure 1:**
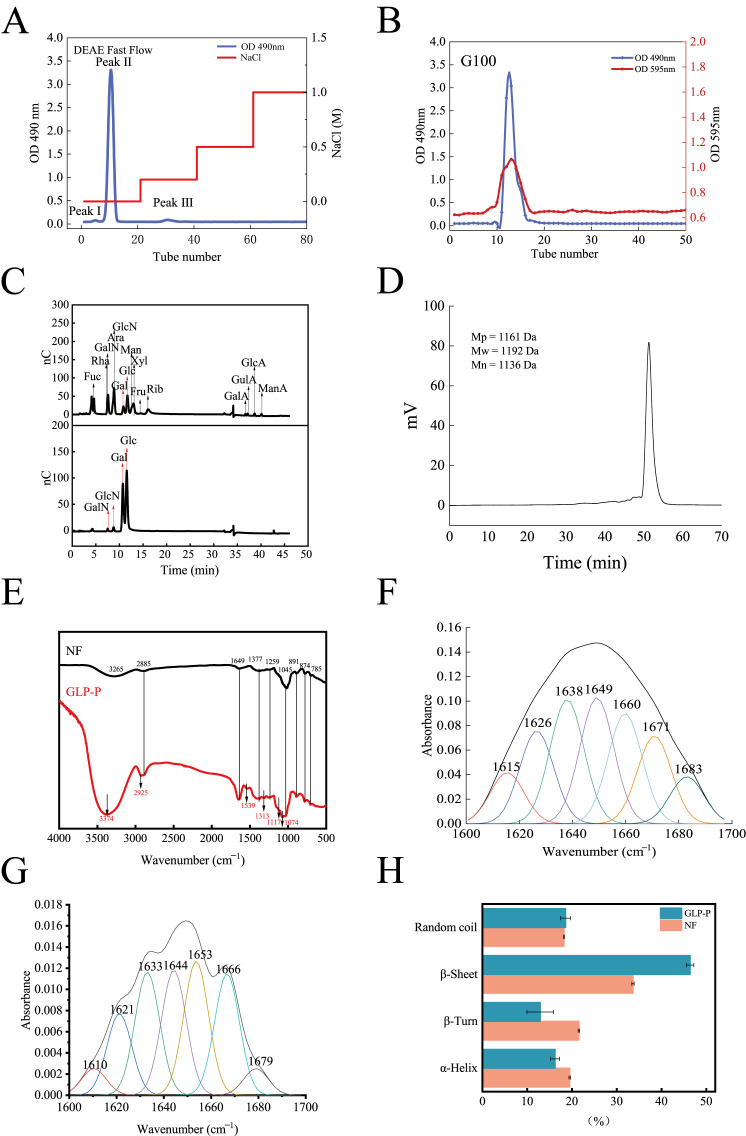
Structural analysis of GLP-P. (**A**) DEAE sepharose Fast Flow elution profile of GLP-P, (**B**) Sephadex G-100 gelelution profile of GLP-P, (**C**) Ion chromatograms of a mixture of 11 monosaccharide standards and GLP-P, (**D**) High performence gel permeation chromatography (HPGPC) information of GLP-P, (**E**) Infrared spectrogram of GLP-P and NF (**F**) Peak fitting curve of GLP-P Amide I band (**G**) Peak fitting curve of NF Amide I band. (**H**) Comparison of secondary structures.

Figure 1A presents the ion-exchange chromatography profile of DEAE Sepharose Fast Flow, showing three distinct elution peaks. Peaks I and II correspond to neutral fractions, whereas Peak III represents an acidic fraction. The recovery rates of the eluted components are summarized in Table 1. Peak II exhibited a narrower peak width and a sharper peak height, suggesting that its components were more concentrated and the elution efficiency was higher. In contrast, Peaks I and III could not be collected in sufficient quantities; therefore, subsequent analyses focused on Peak II.

Figure 1B shows the chromatographic profile of Sephadex G-100 gel filtration, in which a single peak was observed, indicating that the protein and carbohydrate components remained integrated during purification. The sugar and protein contents were 68.11 ± 0.85% and 30.12 ± 1.35%, respectively, speculating the formation of a glycopeptide complex, hereafter referred to as GLP-P.

### 3.2. Structural Characterization and Analysis

#### 3.2.1. Analysis of Monosaccharides and Amino Acids Composition

Figure 1C shows the monosaccharide composition of GLP-P. The molar ratios of galactose (Gal), glucose (Glc), galactosamine (GalN), and glucosamine (GlcN) were 0.637:0.344:0.007:0.013. Notably, Gal and Glc together accounted for the major of the composition, suggesting that the main chain of GLP-P is primarily composed of these two monosaccharides. Previous studies have indicated that differences in milk components and microbial communities may lead to slight variations in monosaccharide composition [35]. Nevertheless, most polysaccharides in kefir are predominantly composed of Gal and Glc.

The amino acid composition of GLP-P is summarized in Table 2, including lysine, asparagine, proline, serine, threonine, arginine, isoleucine, and glycine. The presence of asparagine, serine, and threonine suggests potential sites for O-glycosylation modification in GLP-P [13].

#### 3.2.2. Molecular Weight Distribution and Infrared Spectroscopy Analysis

The molecular weight distribution reflects the homogeneity of the sample components. As shown in Figure 1D, the elution profile of GLP-P exhibited a distinct, sharp, and symmetrical peak, indicating that the components of GLP-P are relatively uniform. The molecular weight (Mw) of its main component was determined to be 1192 Da, classifying GLP-P as a low-molecular-weight glycopeptide complex.

The FT-IR spectrum of GLP-P, recorded in the range of 4000–400 cm^−1^, is shown in Figure 1E, and the corresponding absorption bands with functional group assignments are summarized in Table 3. Notably, no obvious C–O vibration absorption peak was detected in the region of 1740–1690 cm^−1^, suggesting the absence of glucuronic acid [36], which is consistent with the results of monosaccharide composition analysis.

Figure 1F,G show the fitted results of the amide I band for GLP-P and non-fermented milk (NF). The peaks of the amide I band are as follows: 1615–1638 cm^−1^ and 1680–1700 cm^−1^ indicate β-sheet structures; 1639–1648 cm^−1^ indicates disordered coils; 1649–1660 cm^−1^ indicates α-helix structures; 1660–1679 cm^−1^ indicates β-turns [43]. As shown in Figure 1H, analysis of the amide I band reveals a reduced proportion of α-helix and β-turns in GLP-P compared to NF, accompanied by increased content of β-sheets. This structural rearrangement indicates a shift toward greater rigidity and planar stability in the peptide backbone [44]. The emergence of this band indicates a transition from a disordered to a more ordered conformation. Furthermore, spectral peaks at 1529 and 1313 cm^−1^ correspond to the enhancement of the amide II peak and the shift in the amide III peak, respectively, providing additional evidence for this structural transformation. Signals at 1117 and 1074 cm^−1^ reflect sugar ring vibrations, suggesting coupling between the peptide and carbohydrate regions [45]. Collectively, these spectral findings further support the possibility of a covalent sugar-peptide bond within GLP-P.

#### 3.2.3. Methylation Analysis

Methylation analysis determined the glycosylation linkage patterns of GLP-P. Only two sugar residues were identified in GLP-P (Table 4): 2,3,4,6-Me_4_-Galp (52.2%) and 2,3,6-Me_3_-Glcp (47.8%). The corresponding glycosidic bond types were determined as. The terminal glucose residue 2,3,6-Me_3_-Glcp constitutes 47.8% of GLP-P, representing the second most abundant component. This indicates that the glucan moiety in GLP-P possesses relatively short molecular chains, consistent with the low molecular weight of GLP-P. Therefore, we infer that the primary sugar chain backbone of GLP-P consists of β-1,4-galactosidic and β/α-glucan linkages. Isomers of β- and α-glucans (β/α-glucans) are present within this structure.

#### 3.2.4. NMR Analysis

**Figure 2:**
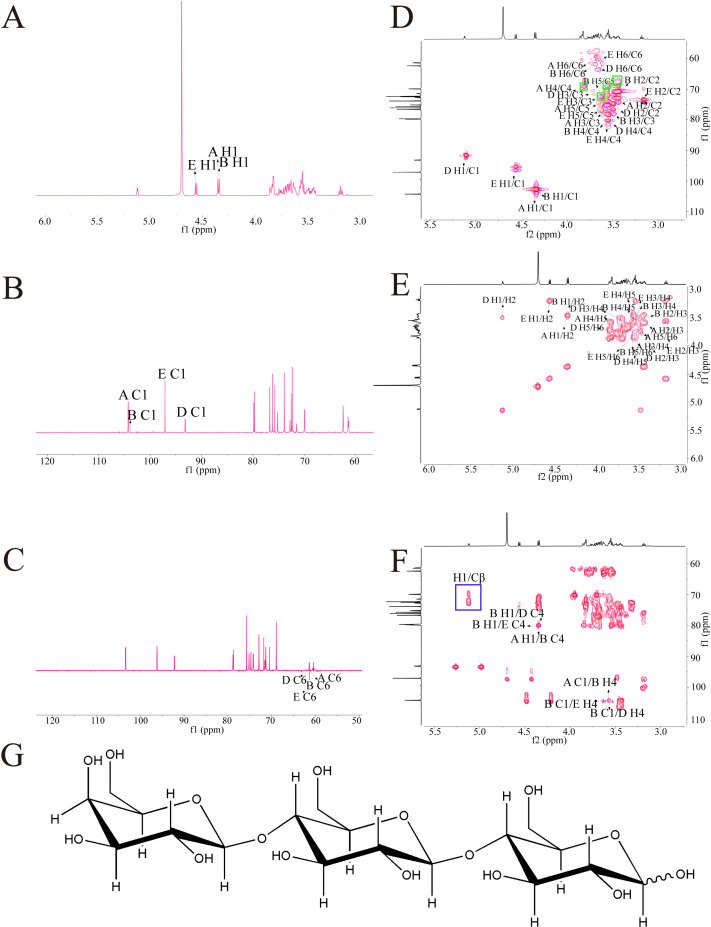
NMR spectra of GLP-P. (**A**) ^1^H NMR spectrum. (**B**) ^13^C NMR spec trum. (**C**) DEPT-135 NMR spectrum. (**D**) HSQC NMR. (**E**) HH-COSY spectrum. (**F**) HMBC. (**G**) Predicted structure of GLP-P.

In this study, the glycosidic bonds in GLP-P were analyzed via ^13^C and ^1^H NMR spectra, with anomeric carbon and proton signals serving as key indicators. In general, α-configurations exhibit anomeric carbon signals at 98–103 ppm and anomeric proton signals at 5.1–5.8 ppm, whereas β-configurations typically show anomeric carbon signals at 103–106 ppm and anomeric proton signals at 4.3–4.8 ppm [46]. Figure 2A illustrates that the proton signals in the hydrogen spectrum of GLP-P were predominantly concentrated between δ3.0–5.5 ppm. At 3.2–4.0 ppm could be assigned to sugar ring protons, which are characteristic of the aliphatic proton environment in glycosidic structures [47]. The main terminal matrix peaks δ5.12, 4.57, 4.35, and 4.34 were concentrated at 4.3–5.5 ppm, indicating that GLP-P contained both α and β configurations. This was consistent with the results of infrared spectrum analysis. By observing the carbon spectrum (Figure 2B), the main nuclear magnetic carbon spectrum signals were concentrated at 60–120 ppm. The chemical shifts in irregular carbons with δ104.27, 104.25, 97.06, and 93.10 in the ^13^C NMR spectrum were mainly in the range of δ93–105. Meanwhile, the δ72.32, 76.63, 70.08, 73.93, 61.45, 72.31, 76.62, 79.85, 73.94, 61.46, 75.72, 72.29, 79.89, 76.22, 62.29, 75.15, 71.50, 79.54, 72.19, and 62.40 main signal peaks were distributed in the region of 60–85 ppm. Our monosaccharide composition analysis indicated that GLP-P was composed of Glu and Gal; hence, we inferred that the saccharide chain of GLP-P was mainly composed of galactoglucan. Additionally, in the Dept-135 spectrum analysis (Figure 2C), the peaks detected at 61.45, 61.46, 62.29, and 62.40 ppm were found to be inverted peaks, indicating the chemical shift of C6. In the HSQC spectrum (Figure 2D), a cross-peak was observed at δ104.27 (anomeric carbon) and δ4.35 (anomeric proton), indicative of a β-anomeric configuration. Through HH-COSY analysis (Figure 2E), sequential proton correlations were identified: H1/H2 (4.35/3.44 ppm) and H2/H3 (3.44/3.56 ppm), marking the proton chemical shifts for H1, H2, and H3 as 4.35, 3.44, and 3.56 ppm, respectively. Based on these correlations and HMBC data (Figure 2F). Table 5 summarises the assignment of all glycosidic bond signals.

In the HMBC spectrum, long-range correlations were observed (Table 6). A correlation signal was observed between the anomeric proton of β-D-Galp-(1→ and the C-4 of β-D-Glcp-(1→. In addition, the anomeric carbon of β-D-Glcp showed correlation signals with the H-4 of both β-D-Glcp and α-D-Glcp, indicating the presence of linkage patterns in which β-D-Glcp is connected via (1→4) glycosidic bonds to either β-D-Glcp or α-D-Glcp. Taken together, these results suggest that the major glycosidic linkage mode of this polysaccharide is β-D-Galp-(1→4)-β-D-Glcp-(1→4)-α/β-D-Glcp, with the coexistence of isomers. The proposed structural formula is shown in Figure 2G.

In the HSQC spectrum (Figure 2D), direct ^1^J-CH correlations of the sugar residue H1/C1 were observed. In addition, signals corresponding to Ser Cβ–Hβ appeared in the region of δ_H 3.5–4.2 ppm and δ_C 65–72 ppm, where a few red cross-peaks were also detected, possibly arising from the –CH–OH group of Thr (highlighted in the green box) [48]. Combined with the HMBC results (Figure 2F), long-range ^2^J/^3^J correlations were detected between the sugar H1 and the peptide side-chain Cβ (highlighted in the blue box), which is feature of O-glycosidic linkage between sugar residues and the hydroxyl carbons of Ser/Thr.

### 3.3. Conformational Analysis

Through the aforementioned experiments, the fundamental composition and glycan structure of GLP-P have been largely elucidated. However, beyond primary structural features, the conformation of glycopeptides profoundly influences their biological activity. Therefore, atomic force microscopy (AFM) and scanning electron microscopy (SEM) were employed to characterize the morphology of GLP-P, with Congo red staining used to confirm its conformational properties.

**Figure 3:**
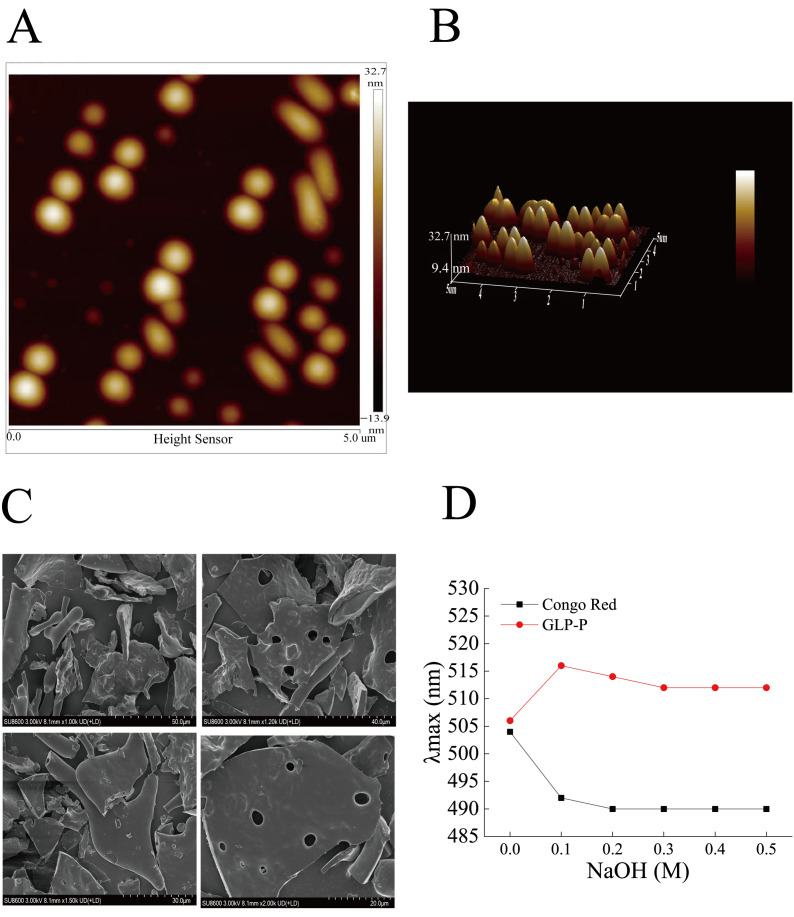
Conformational analysis of GLP-P. (**A**) Atomic force microscopy planar images. (**B**) Atomic force microscopy 3D images. Scale bar = 5 μm. (**C**) SEM images * 1.00 K, * 1.20 K, 1.50 K, 2.00 K. (**D**) Congo red staining of GLP-P.

Figure 3A,B show atomic force microscopy (AFM) images, where GLP-P predominantly exhibits irregular, circular, and elliptical morphologies distributed randomly with varying particle sizes. The height range of GLP-P structures spans from −13.9 to 32.7 nm, forming a multi-island structure. This structural phenomenon likely arises from the aggregation and entanglement of long sugar chains driven by van der Waals forces and other intermolecular interactions [28]. As shown in Figure 3C, the surface morphology of GLP-P includes smooth multilayer structures and rod-like fibers, with sparse pores distributed across the surface. Such layered structures are typically associated with low-molecular-weight polysaccharides [49], while surface pores may increase the material’s contact area with water, thereby enhancing its solubility [50].

In this study, the λmax values of the Congo red-GLP-P complex were measured in alkaline solutions containing varying concentrations of NaOH (0–0.5 M) (Figure 3D). As the sodium hydroxide concentration increased from 0 to 0.1 mol/L, the maximum absorption wavelength of the complex sharply increased from 506 nm to 516 nm, a value significantly higher than that of free Congo red. This red shift suggests the possible presence of ordered regions in GLP-P.

### 3.4. Anti-Inflammatory Activity of GLP-P

#### 3.4.1. Effects of GLP-P on Cytotoxicity

**Figure 4:**
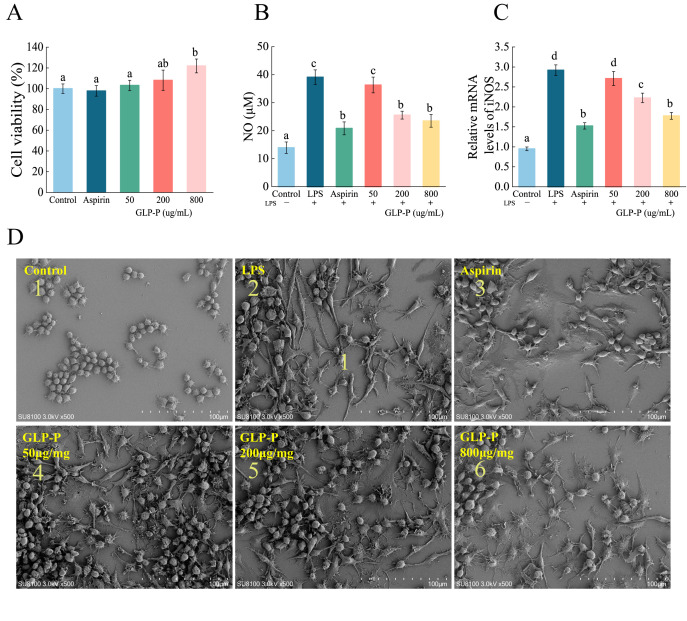
Effects of GLP-P on the Activity of RAW264.7 Cells and LPS-Induced Inflammatory Responses. (**A**) Proliferation rate of RAW264.7 cells. (**B**) NO secretion in RAW264.7 cells. (**C**) mRNA expression levels of iNOS in RAW264.7 cells. Different letters indicate significance between groups, (*p* < 0.05). Data are shown as the mean ± standard deviation (SD), *n* = 3. (**D**) SEM images of the (1) blank control group; (2) LPS positive control group; (3) Aspirin-treated group; (4) 50 μg/mL GLP-P treated group; (5) 200 μg/mL GLP-P treated group; (6) 800 μg/mL GLP-P treated group. Original magnification: ×500.

Macrophages constitute a vital component of the innate immune system, protecting the host against infections and toxins. Furthermore, they regulate key immune responses such as inflammation [51]. Prior to evaluating GLP-P’s anti-inflammatory capacity against LPS-induced inflammation in RAW264.7 macrophages, a CCK-8 assay was conducted to determine optimal sample concentrations and rule out potential cytotoxicity. As shown in Figure 4A, the cell viability of untreated control cells was set as 100%. RAW264.7 macrophages treated with GLP-P (50, 200, and 800 μg/mL) exhibited viabilities of 103.14%, 108.12%, and 121.91%, respectively, after 24 h. Thus, GLP-P enhanced the CCK-8 signal in a dose-dependent manner (*p* < 0.05) within the tested concentration range (50–800 μg/mL). This effect may be associated with the immunomodulatory activity of GLP-P or a mild stimulatory response unrelated to immunomodulation. As the CCK-8 assay measures cellular dehydrogenase activity as an indicator of metabolic state rather than direct proliferation, the observed increase likely reflects enhanced metabolic or enzymatic activity in viable macrophages rather than true cell growth. Importantly, the elevated metabolic activity also indicated the absence of cytotoxicity at the tested concentrations. Based on these results, the range of 50–800 μg/mL was selected for subsequent experiments to evaluate the anti-inflammatory activity of GLP-P in RAW264.7 cells.

When macrophages are stimulated by LPS, they typically exhibit cell spreading, membrane ruffling, and the formation of numerous filopodia and lamellipodia [52]. As shown in Figure 4D, the typical morphology of normal RAW264.7 cells is predominantly round or oval, with uniform size, a smooth surface, and few pseudopodia. Compared to the control group, LPS treatment (Figure 4(D-2)) induced morphological alterations in RAW264.7 cells. Cells became polarized and elongated, exhibiting distinct lamellar structures with prominent folds at the leading edge. The number of pseudopodia markedly increased, transforming from circular to irregular spiny shapes. This pattern resembles findings reported by Galyna et al. [53]. Compared to the LPS group, the aspirin-treated group showed minimal cell elongation and minor morphological alterations. At lower GLP-P concentrations, the irregular cell shapes became increasingly similar to those in the LPS group. At 800 μg/mL GLP-P, the morphology exhibited a more pronounced difference from the LPS group, resembling that of the aspirin group. These results indicate that GLP-P exerts a protective effect on RAW264.7 inflammatory cells.

#### 3.4.2. Effects of GLP-P on LPS-Induced NO Production and iNOS Expression

NO, known as the smallest cellular signaling molecule, is widely recognized as a biomarker of inflammation. Upon activation, macrophages release substantial amounts of nitric oxide (NO), which plays a crucial regulatory role in inflammatory responses. Consequently, inhibiting NO production is commonly employed as a key indicator for evaluating anti-inflammatory activity [54]. LPS (a major tissue-specific antigen) is a potent activator of macrophages [31]. When LPS acts on human and animal cells, these cells exhibit inflammatory responses. Thus, in this study, an inflammatory model was established by stimulating RAW264.7 macrophages with LPS. The effects of GLP-P were evaluated by measuring NO production in these cells. The effect of kefir glycopeptide GLP-P on NO release from inflamed RAW 264.7 cells is shown in Figure 4B. In the control group without LPS, the level of the inflammatory mediator NO secreted by RAW 264.7 cells were low. Compared to the control group, the amount of NO released by RAW 264.7 cells in the LPS group significantly increased (*p* < 0.05), confirming successful induction of the inflammatory model. Compared to the LPS group, NO release progressively decreased with increasing GLP-P concentrations. GLP-P significantly reduced NO production in a dose-dependent manner (*p* < 0.05). At a GLP-P concentration of 200 μg/mL, its ability to inhibit NO release from inflammatory cells showed no significant difference compared to the aspirin group (*p* > 0.05), indicating that GLP-P reduces NO secretion in LPS-induced inflammatory model cells and exerts anti-inflammatory effects in a concentration-dependent manner within a certain range. Since NO production under inflammatory conditions is primarily regulated by iNOS, qRT-PCR was further employed to analyze iNOS mRNA expression levels. As shown in Figure 4C, LPS stimulation significantly upregulated iNOS mRNA expression compared to the control group (*p* < 0.05). The trend in NO release was consistent with the relative expression levels of iNOS mRNA.

#### 3.4.3. The Effect of GLP-P on Cytokine Secretion

**Figure 5:**
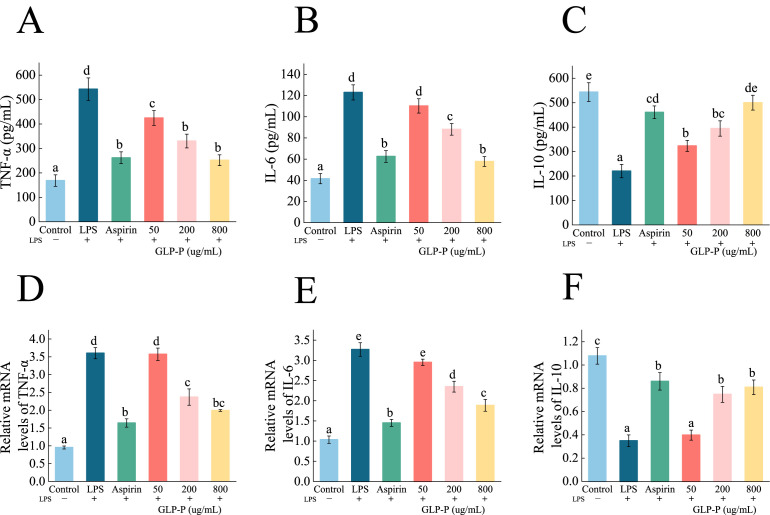
Effect of GLP-P on inflammatory factors in LPS-induced RAW264.7 inflammatory cells. (**A**–**C**) Secretion of (**A**) TNF-α, (**B**)IL-6, (**C**) IL-10 in RAW264.7 cells. (**D**–**F**) mRNA expression levels of (**D**) TNF-α, (**E**) IL-6, and (**F**) IL-10 in RAW264.7 cells. Results are expressed as the mean ± standard deviation (SD), Different letters indicate significance between groups, (*p* < 0.05). Data are shown as the mean ± standard deviation (SD), *n* = 3.

TNF-α and IL-6 are typical proinflammatory cytokines that can trigger inflammatory responses through multiple signaling mechanisms. The emergence of the anti-inflammatory cytokine IL-10 alleviates this inflammatory response. TNF-α and IL-6 are typical proinflammatory cytokines that can trigger inflammatory responses through multiple signaling mechanisms. The emergence of the anti-inflammatory cytokine IL-10 alleviates this inflammatory response. Therefore, maintaining immune homeostasis requires a dual regulatory approach—inhibiting the release of proinflammatory mediators while enhancing the production of anti-inflammatory factors [55,56,57]. The effects of kefir glycopeptide GLP-P on TNF-α and IL-6 secretion by RAW 264.7 inflammatory macrophages are shown in Figure 5A,B. Compared to the control group, LPS-treated cells exhibited significantly increased secretion of the proinflammatory factors TNF-α and IL-6 (*p* < 0.05). Compared to the LPS group, both GLP-P at various doses and aspirin significantly inhibited the secretion of TNF-α and IL-6 cytokines by RAW 264.7 inflammatory cells (*p* < 0.05). With higher GLP-P concentrations yielding greater inhibitory effects. At 800 μg/mL GLP-P, the suppression of TNF-α and IL-6 secretion by inflammatory cells was comparable to that of aspirin (*p* > 0.05). As shown in Figure 5C, LPS significantly reduced IL-10 secretion in RAW264.7 cells (*p* < 0.05). Concurrently, GLP-P treatment significantly increased IL-10 secretion at all tested concentrations (*p* < 0.05). The secretion level in the 800 μg/mL group showed no statistically significant difference compared to the control group (*p* > 0.05). These results indicate that GLP-P exerts a dual anti-inflammatory effect by suppressing pro-inflammatory cytokine production and enhancing anti-inflammatory cytokine secretion, thereby contributing to the restoration of immune balance in inflammatory macrophages.

The anti-inflammatory activity of macrophages is closely associated with changes in the expression of cytokine-related genes. To investigate the immunomodulatory effects of GLP-P at the molecular level, we performed qRT-PCR to evaluate the impact of different GLP-P concentrations on the mRNA expression of inflammatory cytokines in LPS-stimulated RAW264.7 cells. As shown in Figure 5D,E, LPS stimulation significantly elevated mRNA levels of proinflammatory cytokines IL-6 and TNF-α (*p* < 0.05) while markedly suppressing the transcriptional levels of the anti-inflammatory cytokine IL-10 (*p* < 0.05; Figure 5F). Increasing GLP-P concentrations inhibited the expression of proinflammatory factor genes and upregulated the transcription of anti-inflammatory cytokines (*p* < 0.05).

#### 3.4.4. Effects of GLP-P on Protein Levels in the TLR4/MyD88/NF-κB Pathway

**Figure 6:**
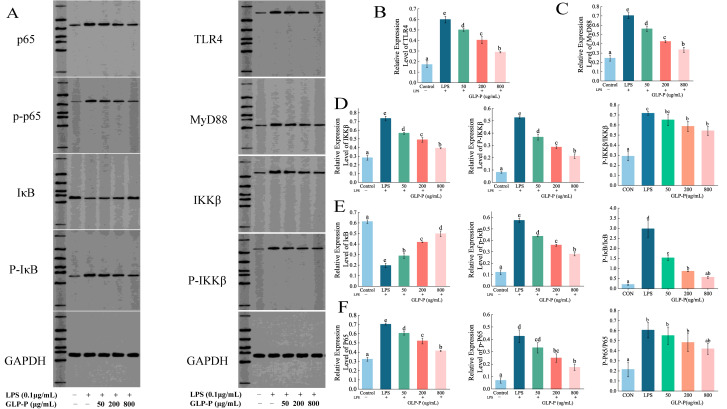
Protein blot analysis of GLP-P in RAW264.7 cells. (**A**) Representative Western blot images showing the expression of TLR4, MyD88, p65, IκB, IKKβ, p-p65, p-IκB, and p-IKKβ. Band intensities were quantified using image analysis software. (**B**) Relative expression of TLR4. (**C**) Relative expression of MyD88. (**D**) Expression of IKKβ, p-IKKβ, and their phosphorylation levels. (**E**) Expression of IκB, p-IκB, and their phosphorylation levels. (**F**) Expression of p65, p-p65, and their phosphorylation levels. Data are presented as mean ± standard deviation (SD), *n* = 3. Different letters indicate statistically significant differences between groups.

Western blot analysis (Figure 6) revealed that LPS stimulation significantly upregulated the protein levels of TLR4, MyD88, and cascade proteins (IKKβ, p-IKKβ, p65, p-p65, p-IκBα), while downregulating IκBα protein levels, showing significant differences compared to the control group (*p* < 0.05), confirming the activation of the signaling pathway. In contrast, GLP-P treatment significantly downregulated TLR4, MyD88, and IKKβ, p-IKKβ, p65, p-p65, and p-IκBα expression in a dose-dependent manner while upregulating IκBα protein levels (*p* < 0.05 compared to the LPS group). To prevent false-positive results, we analyzed the relative phosphorylation levels of IKKβ, IκBα, and p65 (Figure 6D–F). Under these experimental conditions, different doses of GLP-P inhibited the phosphorylation of IκBα. However, inhibiting the phosphorylation of IKKβ and p65 required higher concentrations of GLP-P.

#### 3.4.5. Effects of GLP-P on NF-κB Nuclear Translocation

**Figure 7:**
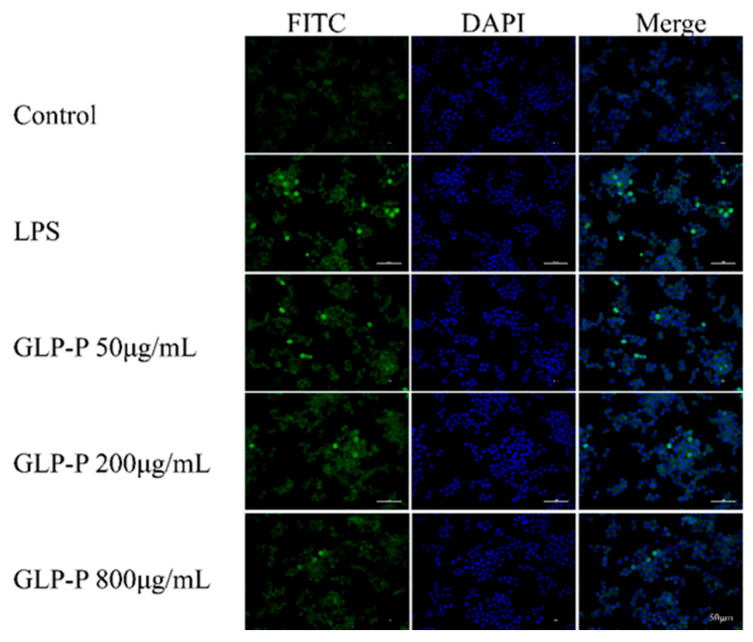
Inhibitory effects of GLP-P on nuclear factor-κB (NF-κB) translocation in RAW264.7 cells. Cells were pretreated with GLP-P (50, 200, or 800 μg/mL) prior to stimulation with LPS (1 μg/mL). The p65 subunit of NF-κB was visualized by green fluorescence, while nuclei were counterstained with DAPI (blue fluorescence). All images are shown at a scale of 50 μm.

**Figure 8:**
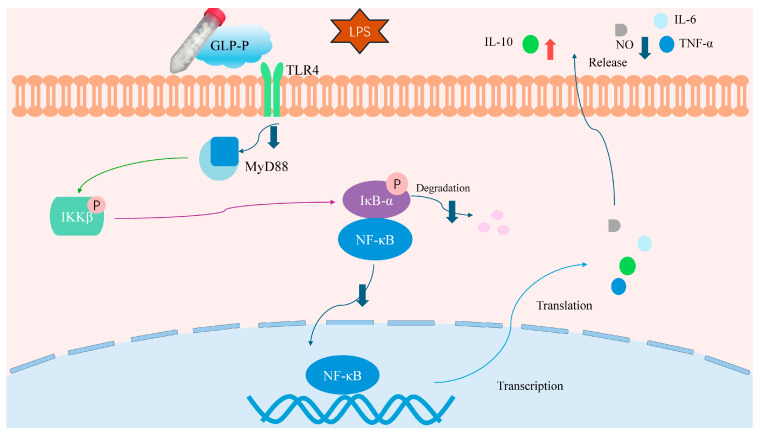
Schematic diagram of GLP-P regulation of immune cells via the TLR4 signaling cascade. GLP-P inhibits the release of pro-inflammatory cytokines and inflammatory mediators induced by LPS through the immune receptor TLR4 and its downstream NF-κB signaling pathway, thereby suppressing inflammation. Abbreviations: GLP-P, kefir-derived glycopeptide; LPS, lipopolysaccharide; TLR4, Toll-like receptor 4; MyD88, myeloid differentiation factor 88; IKK, IκB kinase; NF-κB, nuclear factor-κB; IL-6, interleukin-6; TNF-α, tumor necrosis factor-α; IL-10, interleukin-10; iNOS, inducible nitric oxide synthase; NO, nitric oxide.

To further determine whether GLP-P inhibits NF-κB signaling pathway activation, we employed immunofluorescence to detect NF-κB nuclear translocation. As clearly shown in Figure 7, the green fluorescence areas represent FITC-labeled p65 protein fluorescence, while the blue areas represent DAPI-stained cell nuclei. In the control group, the green fluorescence of p65 was not prominent, and the merged image showed minimal p65 subunits in the nuclei. However, after LPS treatment, the merged image revealed significantly enhanced green fluorescence in the nuclei compared to the control group, indicating substantial nuclear translocation of p65 subunits. Following treatment with different doses of GLP-P, NF-κB p65 nuclear translocation exhibited a dose-dependent, significant reduction. A schematic illustration of the proposed mechanism is shown in Figure 8.

Based on the detection of inflammatory cytokine secretion and mRNA expression, together with immunoblotting and immunofluorescence assays of NF-κB nuclear translocation, those results suggest that GLP-P can alleviate LPS-induced cellular inflammatory responses, possibly through modulation of the TLR4/MyD88/NF-κB signaling pathway in vitro. Specifically: (i) At the transcriptional level, GLP-P downregulated pro-inflammatory genes such as iNOS and TNF-α, while upregulating the anti-inflammatory gene IL-10. (ii) At the signaling pathway level, GLP-P reduced the expression of TLR4, MyD88, and downstream phosphorylated proteins, suggesting suppression of TLR4/NF-κB activation. (iii) At the functional level, GLP-P decreased the release of NO and pro-inflammatory cytokines, while enhancing the secretion of anti-inflammatory mediators. Taken together, these in vitro findings provide preliminary evidence that GLP-P may act as a modulator of inflammatory responses.

## 4. Discussion

Experimental results indicate that GLP-P consists of four monosaccharides and eight amino acids, **t**his suggests that GLP-P may represent a glycopeptide complex, with a molecular weight of approximately 1.1–1.2 kDa. Consistent with previous reports on kefir polysaccharides [35], galactose and glucose are the primary monosaccharide components of GLP-P, with molar ratios of 0.637:0.344. Notably, GLP-P contains asparagine, serine, and threonine residues, which provide potential sites for glycosylation [13]. GLP-P forms near-spherical and ellipsoidal aggregates in aqueous solutions, indicating chain flexibility and a tendency toward random coiling. Internal ordered regions are present, suggesting a locally ordered yet globally flexible conformation. Lysine and proline residues may contribute to enhancing GLP-P’s water solubility and ordered structural stability [58,59]. Research indicates that flexible molecular chains typically exhibit greater height than rigid chains [60], facilitating receptor binding and functional efficacy. Zhang et al. [61] confirmed that flexible conformations may enhance interactions with cell surface receptors, thereby supporting anti-inflammatory effects. Lee’s [62] research indicates that low-molecular-weight polysaccharides exhibit superior antioxidant capacity and immunomodulatory activity compared to high-molecular-weight polysaccharides. This advantage stems from their enhanced solubility and consequently higher bioavailability [63].

Spectroscopic analysis suggests the possible presence of long-range coupling at the O-glycosylation site of GLP-P; however, further confirmation using advanced techniques such as mass spectrometry is required. Recent studies indicate O-glycosylated glycopeptides possess multiple potential physiological functions. Research shows that the O-glycan chain (salicylate-modified) of casein glycomacropeptide (CGMP) enhances intestinal mucus-secretion and improves barrier function [64]. Shiitake mushroom glycoproteins containing O-glycopeptide bonds positively influence the half-life of antioxidant substances [65]. Honey glycopeptides reduce ROS production and significantly interfere with innate immune system molecules in RAW264.7 cells [66], while lycium barbarum glycopeptides suppress inflammatory mediators via the NF-κB pathway in the RAW264.7 model [16]. Ganoderma lucidum glycopeptides markedly inhibit NO, iNOS, and COX-2 expression in RAW264.7 cells [14]. GLP-P was observed to inhibit the release of pro-inflammatory factors such as NO and TNF-α from inflammatory cells, while promoting the secretion of the anti-inflammatory factor IL-10, consistent with changes at the mRNA transcription level. Furthermore, GLP-P appeared to suppress the activation of the TLR4/MyD88/NF-κB pathway in vitro. Collectively, these results provide preliminary evidence that GLP-P may exert anti-inflammatory effects.

In summary, the preliminary findings on the anti-inflammatory activity of GLP-P are encouraging, yet several limitations must be acknowledged. In this study, we evaluated GLP-P only through in vitro assays using an LPS-induced inflammation model, with aspirin as the reference anti-inflammatory compound. Future work should extend to multiple cell models, animal experiments, and comparisons with various reference compounds (e.g., dexamethasone, indomethacin) and other known anti-inflammatory peptides to comprehensively assess its efficacy, dose dependency, safety profile, and in vivo stability. Such investigations will be essential to support its potential application in functional foods and interventions for inflammation-related disorders. Furthermore, regarding the structure–function relationship, it remains necessary to investigate the peptide composition of GLP-P, the presence of potential glycosylation sites, and their impact on its biological effects. Future validation of this structure–activity relationship could be achieved through molecular docking, glycopeptidomics, and in vivo animal studies.

## Figures and Tables

**Table 1:** Elution information.

	Elution Peak Ⅰ	Elution Peak Ⅱ	Elution Peak Ⅲ
DEAE Sepharose Fast Flow	4.78 ± 0.16%	68.98 ± 0.11%	8.83 ± 0.27%
Sephadex G-100	\	85.28 ± 2%	\
Total Yield	\	58.83 ± 1.47%	\

**Table 2:** Amino acid composition.

Amino Acid	Concentration (μg/mg)	Amino Acid	Concentration (μg/mg)
arginine	6.55 ± 0.02	threonine	8.71 ± 0.04
lysine	157.77 ± 0.03	glycine	1.37 ± 0.02
asparagine	30.89 ± 0.01	serine	9.5 ± 0.08
proline	10.89 ± 0.04	isoleucine	1.52 ± 0.04

**Table 3:** Infrared Functional Group Assignment.

Wavenumber (cm^−1^)	Functional Group
3374, 3265	O-H stretching [37]
2925, 2885	C-H stretching [37]
1648	C=O stretching [38] (amide I)
1539	C=O stretching [38] (amide II)
1377	C–H bending [39]
1313, 1259	N–H bending + C–N stretching (Amide III) [40]
1117, 1074	C-O-C Symmetric C–O–C stretching [41]
1045	C-OH [41]
890, 874	C–H bending of α- and β-glycosidic bonds [42]
784	symmetric ring stretching vibration of the pyran ring [30]

**Table 4:** Linkage pattern analysis of GLP-P.

Retention Time (min)	Methylated Sugar	Mass Fragments (*m*/*z*)	Molar Ratio	Type of Linkage
36.910	2,3,4,6-Me_4_-Galp	43, 71, 87, 101, 117, 129, 145, 161, 205	0.522	Galp-(1→
48.409	2,3,6-Me_3_-Glcp	43, 87, 99, 101, 113, 117, 129, 131, 161, 173, 233	0.478	→4)-Glcp-(1→

**Table 5:** ^1^H and ^13^C NMR chemical shifts (ppm) of GLP-P.

Glycosyl Residues	Chemical Shift δ_H/C_ (ppm)						
	H1/C1	H2/C2	H3/C3	H4/C4	H5/C5	H6a/C6	H6b
β-D-Galp-(1→	4.35	3.44	3.56	3.84	3.57	3.67	3.65
A	104.27	72.32	76.63	70.08	73.93	61.45	
→4)-β-D-Glcp-(1→	4.34	3.43	3.53	3.56	3.58	3.68	3.66
B	104.25	72.31	76.62	79.85	73.94	61.46	
→4)-α-D-Glcp	5.12	3.47	3.71	3.54	3.85	3.68	3.62
D	93.10	75.15	71.50	79.54	72.19	62.40	
→4)-β-D-Glcp	4.57	3.17	3.53	3.56	3.50	3.70	3.64
E	97.06	75.72	72.29	79.89	76.22	62.29	

**Table 6:** Glycosidic bond linkage patterns of GLP-P.

X	Y	Glycosidic Bond Linkage Patterns
AH1/BC4	AC1/BH4	β-D-Galp-(1→4)-β-D-Glcp-(1→
BH1/DC4	BC1/DH4	→4)-β-D-Glcp-(1→4)-α-D-Glcp
BH1/EC4	BC1/EH4	→4)-β-D-Glcp-(1→4)-β-D-Glcp

## Data Availability

The original contributions presented in the study are included in the article/Supplementary Material, further inquiries can be directed to the corresponding author.

## Supplementary Materials

The following supporting information can be downloaded at: https://www.mdpi.com/article/10.3390/foods14203509/s1, Table S1: Molecular weight standards; Table S2: Molecular weight standard curve information; Table S3: Monosaccharide composition reference substances; Table S4: Standard monosaccharide samples; Table S5: Amino acid composition reference substances; Table S6: The manufacturer information for Standard amino acid samples; Table S7: ELISA-related reagents and LPS source; Table S8: qPCR primer sequences; Table S9: Western blotting antibody information; Table S10: Fluorescent immunoassay reagent; Figure S1: molecular weight calibration curve.

## Abbreviations

The following abbreviations are used in this manuscript:

GLP-P	kefir glycopeptide
LPS	lipopolysaccharide
TLR4	toll-like receptor 4
NF-κB	nuclear factor-κB
IL-6	interleukin-6
TNF-α	tumor necrosis factor-α
IL-10	interleukin-10
iNOS	inducible nitric oxide synthase
NO	nitric oxide.
IκB	inhibitor of NF-κB
IKK	IκB kinase
MyD88	myeloid differentiation factor 88
