# Supplementary material for: Structural Properties and Anti-Inflammatory Activity of GLP-P, a Kefir-Derived Neutral Glycopeptide
Source: Foods. 2025 Oct 15;14(20):3509. doi: 10.3390/foods14203509 (PMC12563231; doi:10.3390/foods14203509)
Supplement: Supplementary file 1 [file foods-14-03509-s001.zip › foods-3879824-supplementary.pdf]

Table S1. Molecular weight standards

Standard（Da）	Manufacturer	Batch number	Purity
P5	SHOWA DENKO	10202	99%
P10	SHOWA DENKO	10202	99%
P20	SHOWA DENKO	10202	99%
P50	SHOWA DENKO	10202	≥97%
P100	SHOWA DENKO	10202	≥99%
P200	SHOWA DENKO	10202	≥99%
P400	SHOWA DENKO	10202	＞98%
P800	SHOWA DENKO	10202	98%

Table S2. Molecular weight standard curve information

	P5	P10	P20	P50	P100	P200	P400	P800
RT（min）	45.146	43.432	40.483	37.513	34.139	31.719	30.203	28.523
Mp	6902	10610	21750	46520	110000	218500	409000	782700
Mw	7380	10510	21950	44520	109900	217800	410200	755900
Mn	6797	10320	21670	43470	108600	213200	402500	718200
LgMp	3.84	4.03	4.34	4.67	5.04	5.34	5.61	5.89
LgMw	3.87	4.02	4.34	4.65	5.04	5.34	5.61	5.88
LgMn	3.83	4.01	4.34	4.64	5.04	5.33	5.6	5.86

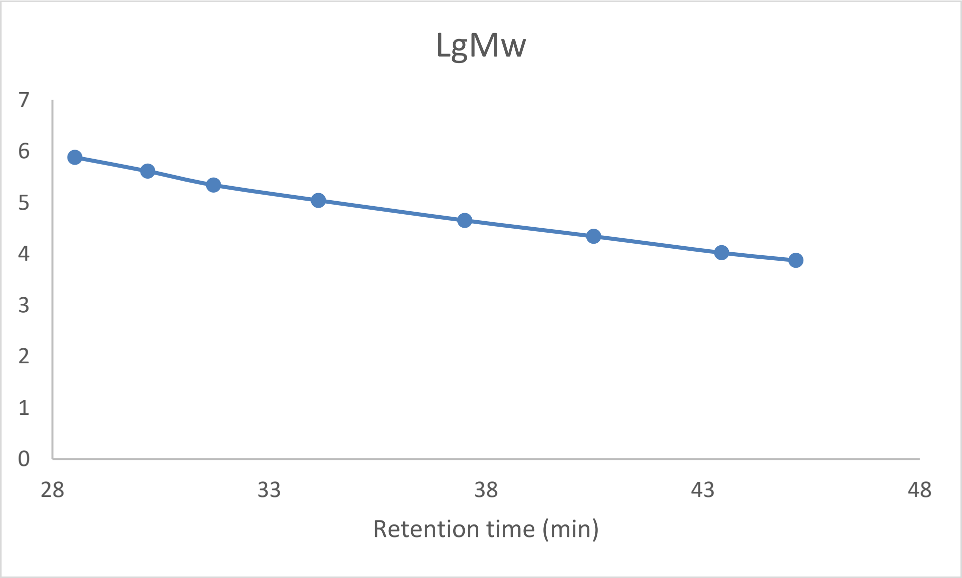

Figure S1 molecular weight calibration curve

Table S3. Monosaccharide composition reference substances

NO	Name	Name (Abbreviation)	Concentration (mg/L)	RT	Area
1	Fucose	Fuc	5	4.608	9.25
2	Rhamnose	Rha	5	7.475	3.927
3	Galactosamine Hydrochloride	GalN	3	7.592	13.87
4	Arabinose	Ara	3.75	8.667	6.567
5	Glucosamine Hydrochloride	GlcN	5	8.858	23.802
6	Galactose	Gal	5	10.842	8.451
7	Glucose	Glc	5	11.692	21.724
8	Mannose	Man	5	12.733	6.242
9	Xylose	Xyl	5	12.983	14.338
10	Fructose	Fru	15	14.525	1.104
11	Ribose	Rib	10	16.083	11.829
12	Galacturonic Acid	GalA	5	36.725	0.871
13	Guluronic acid	GulA	10	37.208	1.179
14	Glucuronic Acid	GlcA	5	38.642	1.641
15	Mannuronic acid	ManA	10	40.108	1.555

Table S4. Standard monosaccharide samples

Standard sample	Manufacturer	Lot Number	Purity
Mannose	Boruitang Biotechnology	C17D9H77586	98%
Rhamnose	Boruitang Biotechnology	H10S9Z69863	98%
Galacturonic acid	Boruitang Biotechnology	K02A9B66077	97%
Galactose	Boruitang Biotechnology	E1927035	99%
Glucose	Boruitang Biotechnology	Q18F10N80946	99%
Glucuronic acid	Boruitang Biotechnology	K14M10S82777	≥98%
Arabinose	Boruitang Biotechnology	S15A10G85850	98%
Xylose	Boruitang Biotechnology	A22S6X3606	99%
Fucose	Boruitang Biotechnology	X29D7Y27768	98%
Glucosamine Hydrochloride	Boruitang Biotechnology	A22S6X3606	98%
D-Fructose	Boruitang Biotechnology	J01J10R89818	99%
D-Ribose	Boruitang Biotechnology	H26F10Z81556	99%
Galactosamine hydrochloride	Boruitang Biotechnology	B01J8S37079	98%
L-Galacturonic acid	Boruitang Biotechnology	S200115AG1	≥98%
D-Mannuronic acid	Boruitang Biotechnology	S200108AM1	≥98%

Table S5. Amino acid composition reference substances

NO	Name	Concentration(mg/L)	RT	Area
1	Arginine	5	1.92	82.104
2	Lysine	5	2.31	64.146
3	Glutamine	5	2.51	22.381
4	Asparagine	5	2.6	24.85
5	Alanine	5	2.82	193.212
6	Threonine	5	2.94	85.377
7	Glycine	5	3.15	271.437
8	Valine	5	3.52	62.965
9	Serine	5	3.97	25.234
10	Proline	5	4.18	16.451
11	Isoleucine	5	4.32	107.642
12	Leucine	5	6.87	134.782
13	Methionine	5	12	79.802
14	Histidine	5	22.37	11.669
15	Phenylalanine	5	22.52	32.06
16	Glutamate	5	22.93	2.489
17	Aspartate	5	23.2	45.2
18	Cysteine	5	23.71	157.766
19	Cystine	5	24.4	46.462
20	Tyrosine	5	24.75	1.091
21	Tryptophan	5	33.36	192.469

Table S6. The manufacturer information for Standard amino acid samples

Standard sample	Manufacturer	Lot Number	Purity
Arginine	Aladdin	J2312087	≥98.5%
Lysine	Aladdin	K2307077	≥98%
Glutamine	Aladdin	E2310118	≥99%
Asparagine	Aladdin	L2312035	≥98%
Alanine	Aladdin	F2330006	≥99%
Threonine	Aladdin	L2324122	≥99%
Glycine	Aladdin	D2328070	≥99%
Valine	Aladdin	H2319057	≥99%
Serine	Aladdin	H2308018	≥99%
Proline	Aladdin	F2314042	≥99%
Isoleucine	Aladdin	J2308140	≥99%
Leucine	Aladdin	H1928149	≥99%
Methionine	Aladdin	E1929099	≥99%
Histidine	Aladdin	G2329005	≥99%
Phenylalanine	Aladdin	D2308030	≥99%
Glutamic acid	Aladdin	J2317054	≥99%
Aspartic acid	Aladdin	C2305089	≥99%
Cysteine	Aladdin	F2313237	≥99%
Cystine	Aladdin	K2322115	≥99%
Tyrosine	Aladdin	D2316045	≥99%
Tryptophan	Aladdin	I1906125	≥99%

Table S7. ELISA-related reagents and LPS source

**Name**	Manufacturer	Product code
Lipopolysaccharide (LPS)	Sigma	L4391-1MG
Mouse Interleukin-10 ELISA Kit	Meimian	MM-0176M1
Mouse Interleukin-6 ELISA Kit	Meimian	MM-0163M1
Mouse Tumor necrosis factor-α ELISA Kit	Meimian	MM-0132M1

Table S8. qPCR primer sequences

Gene	Primer	Sequence (5'-3')	PCR Products
Mus GAPDH	Forward	GAGAGTGTTTCCTCGTCCCGTA	290bp
	Reverse	CCTCACCCCATTTGATGTTAGT	
Mus iNOS	Forward	AGGGAATCTTGGAGCGAGTTG	133bp
	Reverse	GTGAGGGCTTGGCTGAGTGAG	
Mus TNF-α	Forward	AGCACAGAAAGCATGATCCG	212bp
	Reverse	CTGATGAGAGGGAGGCCATT	
Mus IL-6	Forward	CACAGAGGATACCACTCCCAACAGA	124bp
	Reverse	ACAATCAGAATTGCCATTGCACAAC	
Mus IL-10	Forward	GCTGGACAACATACTGCTAACCG	218bp
	Reverse	CACAGGGGAGAAATCGATGACAG	

Table S9. Western blotting antibody information

Antibody name	Manufacturer	Product code
Rabbit polyclonal antibody P65（65KD)	Wuhan Sanying Bioengineering Co., LTD	10745-1-AP
Rabbit polyclonal antibody P-P65 （65KD)	Affinity	AF2006
Rabbit polyclonal antibody IKB（36KD)	ImmunoWay	YT2419
Rabbit polyclonal antibody P-IKB (36KD)	ImmunoWay	YP0151
Rabbit polyclonal antibody TLR4 （100KD)	Affinity	AF7017
Rabbit polyclonal antibody MYD88 (33KD)	Affinity	AF5195
Rabbit polyclonal antibody IKKβ（87KD)	Affinity	AF6010
Rabbit polyclonal antibody P-IKKβ (87KD)	Affinity	AF3010
Rabbit polyclonal antibody GAPDH （37KD）	Hangzhou Xianzhi Biological Co., LTD	AB-P-R 001
HRP-labeled goat anti-rabbit secondary antibody	Wuhan Boshide Bioengineering Co., LTD	BA1054

Table S10. Fluorescent immunoassay reagent

Reagent	Manufacturer	Product code
Goat serum	Wuhan Boshide Bioengineering Co., LTD	AR1009
FITC Goat anti Rabbit IgG（1:400）	Thermo Fisher Scientific	F2765
DAPI	Biyuntian Biotechnology Co., LTD	C1002
NF-κB p65 Polyclonal antibody（1:100）	Wuhan Sanying Bioengineering Co., LTD	10745-1-AP
